# Knowledge, attitude and acceptability of COVID-19 vaccine among residents in rural communities in Ghana: a multi-regional study

**DOI:** 10.1186/s12879-023-08029-x

**Published:** 2023-01-31

**Authors:** Seth Amponsah-Tabi, Rex Djokoto, Stephen Opoku, Ebenezer Senu, Derrick Kyei Boakye, Wisdom Klutse Azanu, Frank Ankobea-Kokroe, Gerald Owusu-Asubonteng, Richard Owusu Ansah, Emmanuel Owusu, Emmanuel Ackah-Avoh, Afia Agyapomaa Kwayie, Eric Appiah Boateng, Richard Pul Azavil, Frederick Ennin

**Affiliations:** 1 Department of Obstetrics and Gynaecology, Komfo Anokye Teaching Hospital, Kumasi, Ghana; 2 Department of Obstetrics and Gynaecology, School of Medicine and Dentistry, Kwame Nkrumah University of Science and Technology, Kumasi, Ghana; 3 Department of Medical Diagnostics, College of Health Sciences, Kwame Nkrumah University of Science and Technology, Kumasi, Ghana; 4 Department of Molecular Medicine, College of Health Sciences, Kwame Nkrumah University of Science and Technology, Kumasi, Ghana; 5 Department of Obstetrics and Gynaecology, University of Health and Allied Sciences, Ho, Ghana; 6 Laboratory Department, Nyaho Medical Center, Greater Accra Region, Accra, Ghana; 7 Laboratory Department, Tamale Teaching Hospital, Northern Region, Tamale, Ghana; 8 Laboratory Department, Bibiani Government Hospital, Western North Region, Bibiani, Ghana

**Keywords:** COVID-19 vaccine, Acceptability, Knowledge, Attitude, Perception

## Abstract

**Background:**

The Coronavirus Infectious Disease 2019 (COVID-19) pandemic has continuously affected human life with several devastating effects. Currently, there are effective vaccines to protect people from COVID‐19 and the World Health Organization (WHO) has highlighted strategies to influence COVID-19 vaccine uptake in hard-to-reach communities in Ghana. However, prior studies on COVID-19 vaccine acceptability in Ghana are online surveys targeting the literates and those in urban areas, leaving residents in far-flung communities. We assessed knowledge, attitude and acceptability of COVID-19 vaccine among residents in rural communities in Ghana.

**Methods:**

This study was a community-based cross-sectional study and was conducted at three selected regions in Ghana (Northern, Ashanti and Western North) from May to November, 2021. This study included residents 15–81 years, living in the selected rural communities for more than 1 year. Study participants were recruited and questionnaires administered to collect data on knowledge, attitude and acceptance of the COVID-19 vaccine. Statistical analyses were performed using Statistical Package for Social Science (SPSS) version 26.0 and GraphPad Prism Version 8.0 software.

**Results:**

Of the 764 participants included in this study, more than half had inadequate knowledge (55.0%), poor attitudes (59.4%) and bad perception about COVID-19 vaccine (55.4%). The acceptability of COVID-19 vaccine in this study was 41.9%. The acceptability of COVID-19 vaccine in Ashanti, Northern and Western North regions were 32.5%, 26.2% and 29.6% respectively. In a multivariate logistic regression analysis, receiving recent or previous vaccine such as HBV vaccine [aOR = 1.57, 95% CI (1.23–3.29), *p* = 0.002], having good attitude towards COVID-19 vaccine [aOR = 61.47, 95% CI (29.55–127.86), *p* < 0.0001] and having good perception about the COVID-19 vaccine [aOR = 3.87, 95% CI (1.40–10.72), *p* < 0.0001] were independently associated with higher odds of accepting COVID-19 vaccine.

**Conclusion:**

More than half of residents in Ghanaian rural communities have inadequate knowledge, poor attitudes and bad perception about COVID-19 vaccine. The acceptability of COVID-19 vaccine is generally low among rural residents in Ashanti, Northern and Western North regions of Ghana. Residents living in hard-to-reach communities must be educated about the benefits of COVID-19 vaccine to achieve effective vaccination program.

## Introduction

The COVID-19 pandemic is continuously harming human life with several devastating effects since the past two years. As of August 26, 2021, there were 215,685,565 confirmed cases of COVID-19 reported globally with 17,978,370 being actively infected and 4,501,003 deaths [1], concurrently, Ghana had recorded 116,441 confirmed cases with 6981 active infections and 991 deaths [1]. Several interventions, such as mandatory wearing of face masks, and social distancing were put in place to fight the pandemic. Unfortunately, the global morbidity and mortality of COVID-19 remains high because of the emergence of new variants and the lack of definitive treatment of the disease and vaccination is the current hope to end COVID-19 [2, 3].

Ghana began its national vaccination program in March 1, 2021, using the Oxford-AstraZeneca vaccine and as of 30th April 2021, Ghana had vaccinated 849,527 people in the first batch [4]. People in the first batch included; healthcare workers, front liners, government officials, the military and people with known underlying conditions. As of November 17, 2022, 28.3% of the total Ghanaian population had vaccinated against COVID-19 [4]. The vast majority of Ghanaians, especially those living in rural areas are yet to take COVID-19 vaccine but there is paucity of data on the knowledge, attitudes and acceptability of COVID-19 vaccine among rural citizens. The WHO have highlighted strategies to drive COVID-19 vaccine uptake in hard-to-reach communities in Ghana [5].

There have been several reports on COVID-19 vaccine acceptability in different countries. Data from 32 countries survey (n = 26,758) on COVID-19 vaccine acceptability range from as low as 38% in Croatia and as high as 98% in Vietnam [6]. Among healthcare workers in the United States, only 36% were willing to take COVID-19 vaccine once it became available [7]. In a Chinese study, 76% students were willing to take COVID-19 vaccine. In this study, students had good attitudes towards the vaccination, however, vaccine uptake was thought to be reduced by alarms about the vaccine safety and efficacy [8]. In a survey among healthcare workers in Saudi Arabia, the acceptability of COVID-19 vaccine was about 50%, alarming for more education to alleviate fear during vaccination [9]. Reports from Bangladesh indicates that majority of the citizens have inadequate knowledge towards COVID-19 vaccine among the general population in Bangladesh [10]. In a recent study among participants in low-middle- and high-income countries in the East Mediterranean Region, the acceptability of booster dose acceptance ranges from 73.4% in low income countries to 80.3% in high-income countries [11]. In a regional study in Africa, a recent study found high level of COVID-19 vaccine hesitancy among medical students in Sudan [12].

In a nationwide online survey, the acceptability was 65% among residents in the 16 regions of Ghana [13]. Although Lamptey et al. [14], and Agyekum et al. [15], spearheaded research into the knowledge, attitudes and acceptability of COVID-19 vaccine in Ghana, Lamptey’s study used google forms as a data collection tool which targeted the educated class thus ignoring the uneducated folks who form a majority of the Ghanaian population. Also, Agyekum’s study featured healthcare workers only; thus, ignoring most of the Ghanaian populace. The vast majority of Ghanaians in rural areas are yet to receive the COVID-19 vaccine and with the aim of implementing the most effective vaccination strategy in Ghana, it is therefore important to examine community’s knowledge, attitude and acceptance of the COVID-19 vaccine. We therefore assessed knowledge, attitude and acceptability of COVID-19 vaccine among residents in rural communities of three-selected regions in Ghana.

## Materials and methods

### Study design and sites

**Fig. 1:**
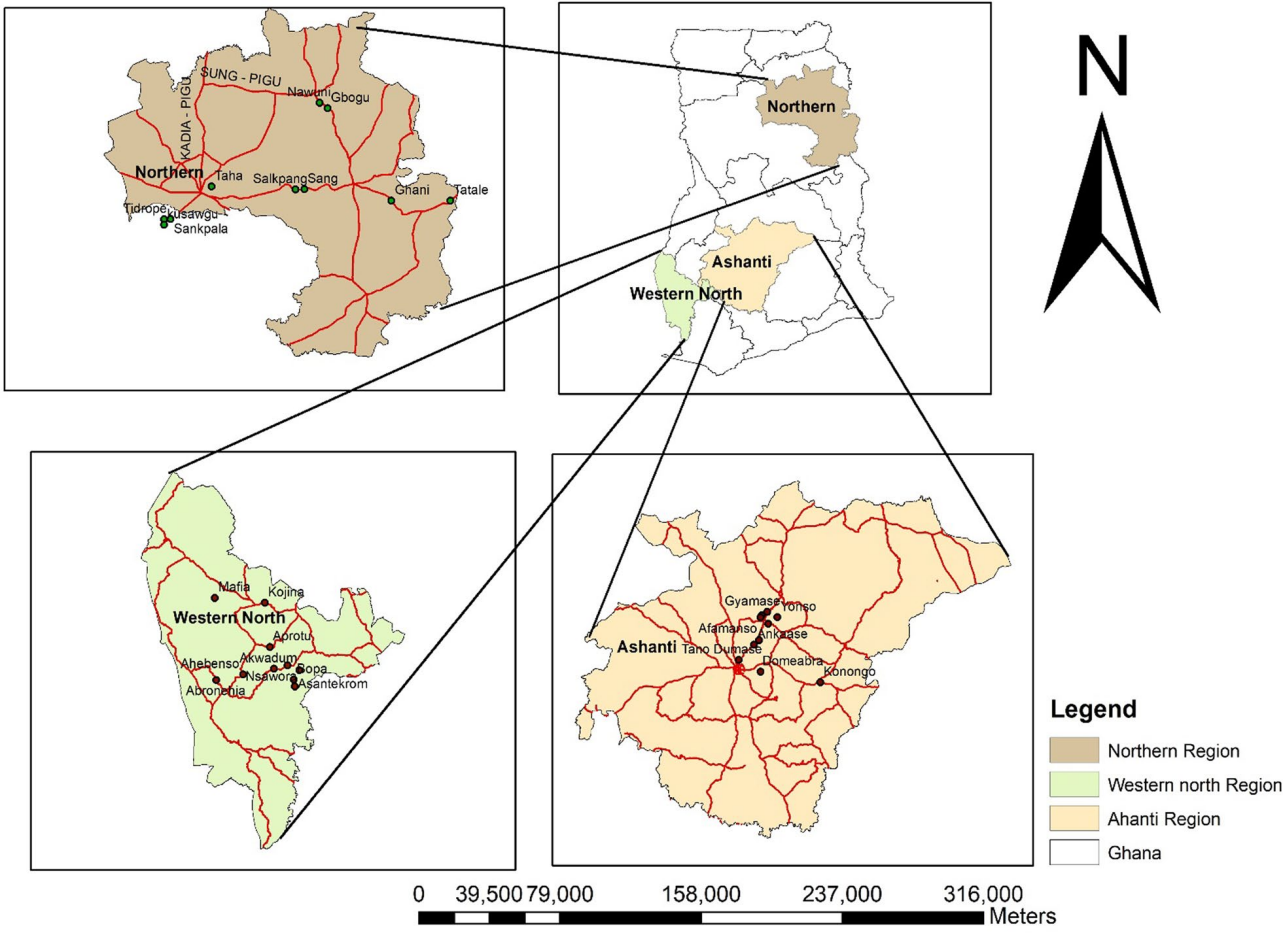
Map of Ghana showing Northern, Ashanti and Western North Regions, and their respective communities included in this study

This study was a community-based cross-sectional study. Study participants were recruited and questionnaires administered to collect data on knowledge, attitude and acceptance of the COVID-19 vaccine. This study was conducted at three-selected regions in Ghana which includes Ashanti, Northern and Western North Regions. In the Ashanti region, 10 communities namely; Bedomasi, Gyamase, Yonso, Akrofonso, Afamanaso, Domeabra, Konongo, Tano- Odumase, Ankaase and Asonomaso-Nkwanta were included. In the Northern region 10 communities were included. These communities are; Nawuni, Gbogu, Sankpala, Tidrope, Kusawagu, Salkpang, Taha, Sang, Tatale and Ghani. In the Western North Region 10 communities were also included. These communities are Betenase, Nsawora, Bopa, Akwadum, Asantekrom, Kojina, Mafia, Aprotu, Abronehia and Ahebenso. Figure 1 displays the map of Ghana showing the three-selected regions and their respective communities included in this study.

### Study population, inclusion/exclusion criteria

This study was conducted among residents of selected rural communities in Ghana, 15–81 years, who have lived in these communities for more than 1 year. However, residents were excluded from the study if they were less than 15 years, had lived in selected communities for less than 1 year, mentally or critically unstable. In addition, residents who failed to sign the consent form or disagreed to participate were excluded from the study.

### Sample size estimation

The sample size was calculated from the Cochran’s formula [16], n = $\frac{{Z}^{2} p.q}{{d}^{2}}$; where n is the minimum sample size required, Z is the z-value at 95% confidence interval (thus, 1.96), *d* is the margin of error (0.05) and *p* is proportion of COVID-19 acceptability in Ghana (54.1%) [14] and *q* is the variability (1-*p*). Substituting these values in the equation gives; $n= \frac{{\left(1.96\right)}^{2}*\left(0.541\right)*(0.459)}{{(0.05)}^{2}}$, n = 381.6. Providing a non-response distribution of 10% (35), the minimum sample size required for this study was 420. To increase statistical power, a total of 764 participants were recruited in this study.

### Ethics approval and consent to participate

Ethical approval was sought from the Committee on Human Research, Publication and Ethics, School of Medical Sciences, Kwame Nkrumah University of Science and Technology (CHRPE/AP/293/22). Prior to the start of the study, written and informed consent was also obtained from study participants or parents/guardians (for participants less than 18 years). Thorough explanation of the study protocol and assurance of anonymity was made to the subjects that they are going to represented by codes rather than their names. All methods were performed in accordance with the relevant guidelines and regulations.

### Data collection technique and tools

A simple random sampling technique was used to sample study participants from the households of communities within the selected regions. In the simple random sampling technique, all households were given numbers according to the Ghana Postal addressing system (GPS). A total of 764 households were selected at random from the GPS application. The researcher was guided then by the addressing system to the selected households. A well-structured, close-ended questionnaire was used as the data collection instrument to gather data from respondents selected for the study. The first section of the questionnaire constituted the respondents’ demographic characteristics whilst the remaining sections were designed based on the study’s specific objectives. Primary data was collected from study participants at the comfort of their homes.

### Classifying knowledge, attitude and perception about COVID-19

To assess the level of knowledge, attitudes, and perceptions of the respondents, a total of 19 items structured questions (including 8-items for knowledge, 6-items for attitudes and 6-items for perceptions). All questions were based on validated questions in previous literature [10, 17]. The knowledge section comprised 8-items with different forms of responses. Some require “Yes” or “No” and others require multiple answer questions where participants have the option to select more than one response. Participants were given scores based on their response to the questions. A correct response attracted a score of 1 and a wrong response attracted a score of 0. The maximum score for knowledge was 12.

The attitude section comprised 6-items which require a “Yes” or “No” response. Participants were given scores based on their response to the questions. A correct response attracted a score of 1 and a wrong response attracted a score of 0. The maximum score for attitude was 6.

The perception section comprised 6-items with different forms of responses. Some require “Yes” or “No” and others require multiple answer questions where participants have the option to select more than one response. Participants were given scores based on their response to the questions. A correct response attracted a score of 1 and a wrong response attracted a score of 0. The maximum score for perception was 10.

Since there is no accepted cutoff for knowledge, attitude and perception about COVID-19 vaccine, the researcher used the threshold of 50% for categorization. Participants were classified as having inadequate knowledge if they had a score of 0–6 and participants that had scores 7–12 were classified as having adequate knowledge. Again, participants were classified as having poor attitude if they had a score of 0–3 and participants that had scores 4–6 were classified as having good attitude.

Moreover, participants were classified as having bad perception if they had a score of 0–5 and participants that had scores 6–10 were classified as having good perception.

### Statistical analysis

Relevant data obtained from respondents was analyzed by employing descriptive and inferential statistics. Data obtained were entered and cleaned in Microsoft Excel 2016. Statistical analyses were performed on Statistical Package for Social Science (SPSS) version 25.0 software and GraphPad Prism Version 8.0. All the study variables were categorical and were therefore presented by frequencies and percentages. The Chi-square test was performed to establish the factors associated with COVID-19 vaccine acceptability. The univariate and multivariate logistic regression were used to asses for independent predictors of COVID-19 vaccine acceptability. *p*-values less than 0.05 were considered as statistically significant for all analyses (Bold).

## Results

### Sociodemographic characteristics of study participants

A total of 764 participants consented and were included in the statistical analysis. More than one-third (38.5%) of the study participants were 21–29 years with the least proportion (7.2%) within 51–59 years. In this study, there was slightly more males (55.9%) than females (44.1%) participants. Majority of the study participants had no formal education or had attended basic education (68.7%) and with 7.2% having tertiary education. The highest proportion of the study participants were single or cohabiting (68.7%), others were married (28.4%) and a few were divorced, widows or widowers (4.7%). Majority of the participants were currently working in the informal sector (56.7%) and the remaining were either working in the formal (19.5%) sector or were unemployed (23.8%). Most of the participants were Christians (82.7%) or living as a nuclear family (84.5%).

Majority of the study participants had a monthly income less than GHS 1000 (73.1%). With respect to participant’s history of chronic conditions, most of them did not have any chronic condition (93.3%). However, a few participants had history of other conditions (6.7%). Of the 25 participants who reported history of chronic conditions, some had asthma (7), diabetes (7), hypertension (6) and others conditions (5). More than half of the study participants have not recently or previously taken any vaccine (55.1%) whiles the remaining participants had recently or previously been vaccinated with other vaccines aside COVID-19 vaccine (44.9%). Table 1 displays the sociodemographic characteristics of the study participants.

**Table 1:** Sociodemographic characteristics of study participants

Variable	Frequency (n = 764)	Percentage (%)
Age category		
15–20	82	10.9
21–29	290	38.5
31–39	102	13.5
41–49	*148*	*19.6*
51–59	54	7.2
60–81	*78*	*10.3*
Sex		
Male	426	55.9
Female	336	44.1
Education		
None/Basic	514	68.7
JHS/SHS	180	24.1
Tertiary	54	7.2
Marital Status		
Married	216	28.4
Single/cohabiting	508	66.8
Divorced, widow or widower	36	4.7
Employment		
Formal	146	19.5
Unemployed	178	23.8
Informal	424	56.7
Religion		
Christian	630	82.7
Muslim	90	11.8
Traditionalist	20	2.6
No affiliation	22	2.9
Ethnicity		
Akan	384	50.4
Northerner	78	10.2
Ga Dangme/Ewe	48	6.3
Other	252	33.1
Family type		
Nuclear	634	84.5
Extended family	116	15.5
Income (GH₵)		
< 1000	446	73.1
1000–2000	144	23.6
> 2000	20	3.3
Chronic condition		
No	700	93.3
Yes	50	6.7
Specify (n = 50)		
Asthma	14	28.0
Diabetes	14	28.0
Hypertension	12	24.0
Other conditions	10	20.0
Ever received vaccine when you turned 18 years		
No	412	55.1
Yes	336	44.9

JHS: Junior High School, SHS: Senior High School, HBV: Hepatitis B virus, some variables have missing values

### Knowledge about COVID-19 vaccine

Majority of the study participants knew about the COVID-19 vaccine (92.7%) with most of them having heard about it through radio or TV (65.7%). Moreover, some participants have ever heard about the COVID-19 vaccine through social media (47.6%), internet (34.0%), family (31.2%), friends and neighbors (33.0%). Majority of the study participants believed that it is dangerous to use overdose of the COVID-19 vaccine (74.1%) with 62.3% disagreeing with vaccine being effective. Furthermore, more than half of the study participants were not aware the vaccine can cause serious adverse effects (52.1%). In addition, majority of the study participants responded that the COVID-19 vaccine cannot increase allergic reactions (74.1%) or autoimmune diseases (58.4%) (Table 2).

**Table 2:** Responses to questions on knowledge about COVID-19 vaccine

Question	No [n (%)]	Yes [n (%)]
Do you know about COVID-19 vaccine?	56 (7.3)	708 (92.7)
How did you get to know COVID-19 vaccines first?		
Media (Radio, TV)	262 (34.3)	502 (65.7)
Social media (WhatsApp, Facebook, Twitter, Instagram etc.)	600 (78.5)	164 (21.5)
Internet	604 (79.1)	160 (20.9)
Newspaper	630 (82.5)	134 (17.5)
Family members	526 (68.8)	238 (31.2)
Friends and Neighbors	512 (67.0)	252 (33.0)
Is the COVID-19 vaccine effective?	476 (62.3)	288 (37.7)
Is it dangerous to use overdose vaccines?	198 (25.9)	566 (74.1)
Does vaccination increase allergic reactions?	566 (74.1)	198 (24.9)
Does vaccination increase autoimmune diseases?	446 (58.4)	318 (41.6)
I am aware of serious adverse effects of COVID-19 vaccine	398 (52.1)	366 (47.9)

**Fig. 2:**
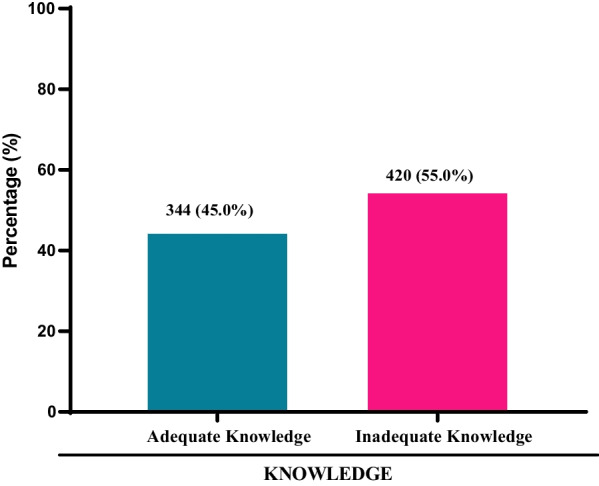
Knowledge of COVID-19 vaccine among study participants

Almost half of the study participants had adequate knowledge about COVID-19 vaccine (45.0%). However, more than half of the study participants had inadequate knowledge about the COVID-19 vaccine (55.0%) (Fig. 2).

### Attitude towards COVID-19 vaccine among study participants

More than half the number of study participants did not agree that the COVID-19 vaccine is safe (57.6%), however a similar proportion stated that the COVID-19 vaccine is essential for public health (51.0%). Majority of the study participants agreed it was not possible to reduce the incidence of COVID-19 without vaccination (63.6%) and that the vaccine should be distributed fairly within the population (64.7%). However, a few of the study participants mentioned they would encourage family, friends and relatives to get vaccinated (36.9%) (Table 3).

**Table 3:** Responses to questions about CVID-19 vaccine attitude

Question	No [n (%)]	Yes [n (%)]
The newly discovered COVID-19 vaccines are safe	440 (57.6)	324 (42.4)
The COVID-19 vaccines are essential for us	374 (49.0)	390 (51.0)
I will take the COVID-19 vaccine without any hesitation, if it is available in Ghana	482 (63.1)	282 (36.9)
I will also encourage my family/friends/ relatives to get vaccinated	442 (57.9)	322 (42.1)
It is not possible to reduce the incidence of COVID-19 without vaccination	278 (36.4)	486 (63.6)
The COVID-19 vaccine should be distributed fairly to all of us	270 (35.3)	494 (64.7)

**Fig. 3:**
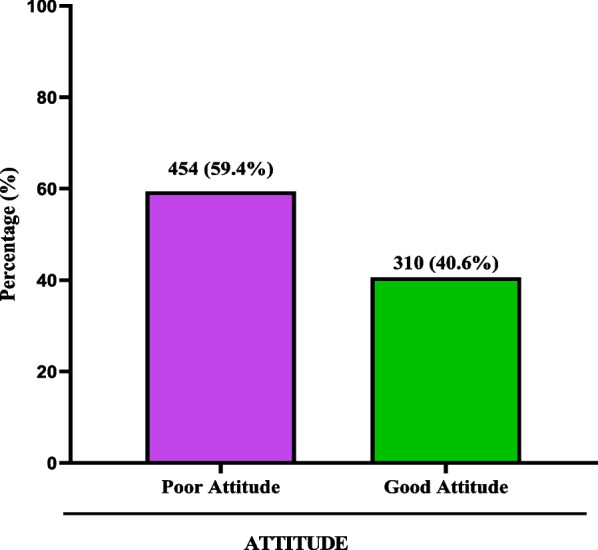
Attitudes towards COVID-19 vaccine among study participants

Acceding to 50% cutoff, majority of the study participants had poor attitudes towards COVID-19 vaccine (59.4%), while the remaining participants had good attitudes towards COVID-19 vaccine (40.6) (Fig. 3).

### Participants perception about COVID-19 vaccine

Majority of the study participants think the newly discovered Covid-19 vaccine may have side effects (67.8%) and most responded that if everyone in the society maintains the preventive measures, the COVID-19 pandemic could be eradicated without vaccination (56.8%). When participants asked about their perception on who should have been vaccinated, majority of the participants mentioned those who have never been infected with COVID-19 (85.1%), COVID-19 infected patients (70.4%), the newly recovered (83.8%) or should be distributed to everyone (50.0%). In addition, most of the study participants indicated they would not buy the vaccine at their own expense if the government do not provide it for free (74.6%). Regardless, majority of the participants thought the vaccine should be administered free of charge in Ghana (79.8%) (Table 4).

**Table 4:** Responses to questions about CVID-19 vaccine attitude

Question	No [n (%)]	Yes [n (%)]
Do you think the newly discovered COVID-19 vaccine may have side effects?	246 (32.2)	518 (67.8)
Do you think that if everyone in the society maintains the preventive measures, the COVID-19 pandemic can be eradicated without Vaccination?	330 (43.2)	434 (56.8)
Who should have been vaccinated, what do you think?		
Never infected	114 (14.9)	650 (85.1)
COVID-19 infected patients	226 (29.6)	538 (70.4)
Newly recovered	124 (16.2)	640 (83.8)
Everyone	382 (50.0)	382 (50.0)
Do you have any religious or spiritual perception concerning the COVID-19 vaccine?	574 (75.1)	190 (24.9)
Do you think the vaccine should be administered free of charge in Ghana?	154 (20.2)	610 (79.8)
Would you buy the vaccine at your own expense if the government did not provide it free?	570 (74.6)	194 (25.4)

**Fig. 4:**
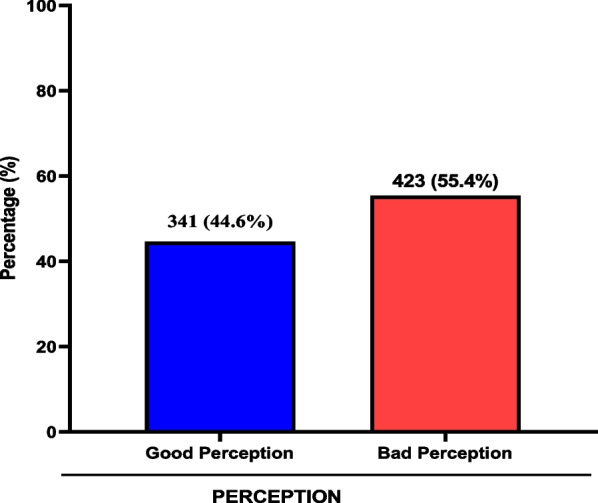
Perception about COVID-19 vaccine among study participants

Majority of the study participants had a bad perception about COVID-19 vaccine (55.4%). On the other hand, some participants had good perception about the COVID-19 vaccine (44.6%) (Fig. 4).

### COVID-19 vaccine acceptability among study participants

**Fig. 5:**
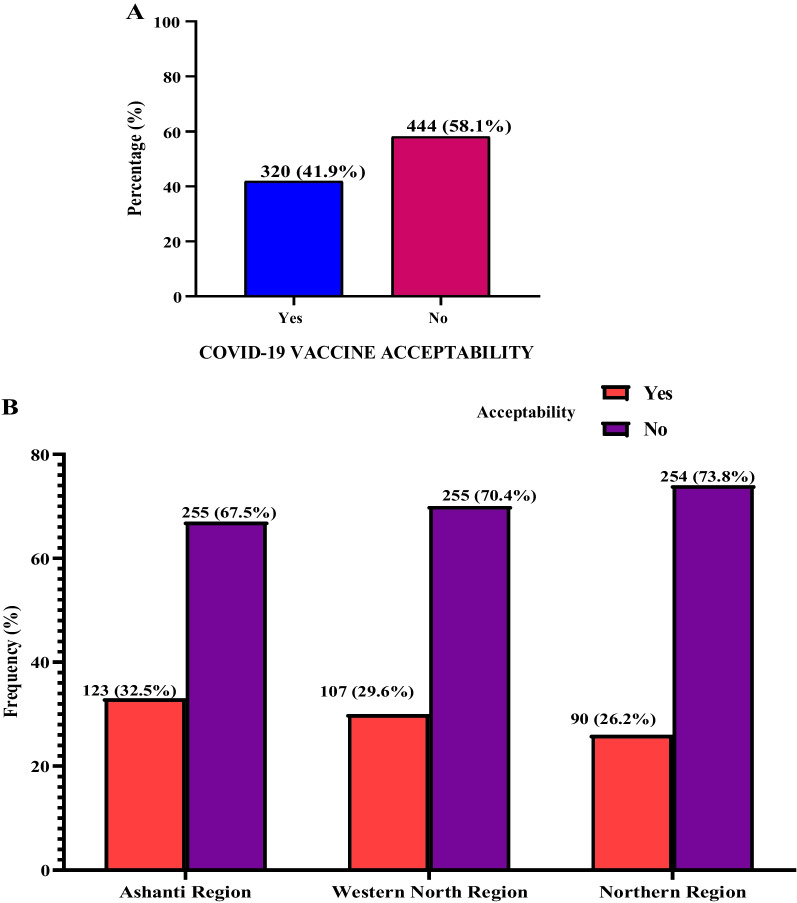
Acceptability of the COVID-19 vaccine among study participants

When participants were asked about their willingness to accept the COVID-19 vaccine, more than half of the participants replied they would not accept the vaccine (58.1%). The acceptability of COVID-19 vaccine in this study was 41.9% (Fig. 5A). The acceptability of COVID-19 vaccine in Ashanti, Northern and Western North regions were 32.5%, 26.2% and 29.6% respectively (Fig. 5B).

### Factors associated with COVID-19 vaccine acceptability

We assessed the putative factors associated with COVID-19 vaccine acceptability among the study participants. We observed that the educational level (*p* = 0.013), employment status (*p* = 0.026) and receiving recent or previous vaccine such as HBV vaccine (*p* < 0.0001) were significantly associated with COVID-19 vaccine acceptability (Table 5).

**Table 5:** Factors associated with COVID-19 vaccine acceptability

COVID-19 vaccine acceptability			
Variable	No (n = 444)	Yes (n = 320)	*p*-value
Age category			
15–20	48 (11.0)	34 (10.7)	0.495
21–29	156 (36.2)	132 (41.5)	
31–39	72 (16.5)	30 (9.4)	
41–49	84 (19.3)	64 (20.1)	
51–59	32 (7.3)	22 (6.9)	
60–81	42 (9.7)	36 (11.3)	
Sex			
Male	252 (56.8)	174 (54.7)	0.693
Female	192 (43.2)	144 (45.3)	
Education			
None/Basic	282 (64.7)	232 (74.4)	**0.013**
JHS/SHS	120 (27.5)	60 (19.2)	
Tertiary	34 (7.8)	20 (6.4)	
Marital			
Married	228 (25.8)	102 (32.1)	0.268
Single or cohabiting	310 (70.1)	198 (62.2)	
Divorced, widow or widower	18 (4.1)	18 (5.7)	
Employment			
Formal	72 (16.4)	74 (23.9)	**0.026**
Unemployed	124 (28.3)	54 (17.4)	
Informal	242 (55.3)	182 (58.7)	
Religion			
Christian	366 (82.4)	264 (83.0)	0.745
Muslim	52 (11.7)	38 (11.9)	
Traditionalist	10 (2.3)	10 (3.2)	
No affiliation	16 (3.6)	6 (1.9)	
Ethnicity			
Akan	216 (48.9)	168 (52.5)	0.414
Northerner	46 (10.4)	32 (10.0)	
Ga Dangme/Ewe	22 (5.0)	26 (8.1)	
Other	158 (35.7)	94 (29.4)	
Family type			
Nuclear	374 (85.0)	260 (83.9)	0.766
Extended	66 (15.0)	50 (16.1)	
Income			
< 1000	262 (75.3)	184 (70.2)	0.429
1000–2000	78 (22.4)	66 (25.2)	
> 2000	8 (2.3)	12 (4.6)	
Chronic condition			
No	410 (94.5)	290 (91.8)	0.301
Yes	24 (5.5)	26 (8.2)	
Ever received vaccine			
No	292 (67.0)	120 (38.5)	**< 0.0001**
Yes	144 (33.0)	192 (61.5)	

### Predictors of COVID-19 vaccine acceptability among the study participants

In a univariate logistic regression model, working in the informal sector was significantly associated with lesser odds of accepting the COVID-19 vaccine [cOR = 0.42, 95% CI (0.22–0.80), *p* = 0.009]. However, having tertiary education [cOR = 1.40, 95% CI (1.02–3.17), *p* = 0.045], recent or previous vaccination [cOR = 3.24, 95% CI (2.11–4.98), *p* < 0.0001] and adequate knowledge about COVID-19 vaccine [cOR = 1.61, 95% CI (1.07–2.43), *p* = 0.023] increased the odds for accepting the vaccine. Similarly, good attitude towards COVID-19 vaccine [cOR = 67.70, 95% CI (35.11–129.79), *p* < 0.0001] and good perception about the COVID-19 vaccine [cOR = 9.67, 95% CI (4.83–19.36), *p* < 0.0001] were significantly associated with higher odds of accepting the COVID-19 vaccine.

After adjusting for possible confounders in a multivariate logistic regression model, recent or previous vaccination [aOR = 1.57, 95% CI (1.23–3.29), *p* = 0.002], good attitude towards COVID-19 vaccine [aOR = 61.47, 95% CI (29.55–127.86), *p* < 0.0001] and good perception about the COVID-19 vaccine [aOR = 3.87, 95% CI (1.40–10.72), *p* < 0.0001] were the independent predictors of COVID-19 vaccine acceptability (Table 6).

**Table 6:** Multivariate analyses of predictors of COVID-19 vaccine acceptability

Variable	aOR (95% CI)	*p-*value
Education		
None/Basic (Ref)	1.00	–
JHS/SHS	0.48 (0.09–2.54)	0.389
Tertiary	0.66 (0.12–3.52)	0.623
Employment		
Formal (Ref)	1.00	–
Informal	0.61 (0.16–2.33)	0.467
Unemployed	0.95 (0.35–2.56)	0.923
Recent or Previous vaccination (e.g.: HVB, Polio etc.)		
No (Ref)	1.00	–
Yes	1.57 (1.23–3.29)	**0.002**
Vaccine knowledge		
Inadequate (Ref)	1.00	–
Adequate	1.74 (0.54–2.42)	0.736
COVID-19 vaccine attitude		
Bad attitude (Ref)	1.00	–
Good attitude	61.47 (29.55–127.86)	**< 0.0001**
Vaccine perception		
Bad perception (Ref)	1.00	–
Good perception	3.87 (1.40–10.72)	**< 0.0001**

Bold: statistically significant at *p* < 0.05. Model was adjusted for age, sex, income, religion, ethnicity, marital status, family type, residence and the presence of other conditions

## Discussion

The COVID-19 pandemic is continuously harming human life with several devastating effects since the past year. Currently, vaccines are the effective strategy to protect the population from COVID-19, since SARS‐CoV‐2 is highly contagious virus and affects populations widely and globally. Prior studies assessing COVID-19 vaccine acceptability in Ghana are online surveys targeting the literates and those in urban areas, leaving residents in far-flung communities.

In this study, more than half of the study participants had inadequate knowledge (55.0%), poor attitudes (59.4%) and bad perception about COVID-19 vaccine (55.4%). The acceptability of COVID-19 vaccine in this study was 41.9%. The acceptability of COVID-19 vaccine was generally low across all the three-selected regions in Ghana. In a multivariate logistic regression model, receiving recent or previous vaccine such as HBV vaccine, having good attitude towards COVID-19 vaccine and having good perception about the COVID-19 vaccine were independently associated with higher chances of accepting COVID-19 vaccine.

In the current study, the acceptability of COVID-19 vaccine was 41.9%. Our finding is comparable to the study of Agyekum et al. [18], who reported that 39.3% of health care workers intended to receive the COVID-19 vaccines. However, our study finding is much lower compared to studies of Alhassan et al. [19] and Acheampong et al. [20]*,* who found about half of health care workers and mostly urban adult Ghanaians over 15 years were likely to take the COVID-19 vaccine if made generally available. Similarly, our study finding is much lower compared to studies by Wang et al. [21], and Bell et al. [22], who reported COVID-19 vaccine acceptance rate of about two-third 73.31% and more than half 55.8% respectively among the Chinese. The difference observed in our study compared to previous studies may be due to our study conducted in the rural and hard-to-reach communities in Ghana in contrast to previous studies that targeted the literates such as health workers and individuals living in the urban areas who might have had some education about vaccination. This calls for the need to enhance vaccine education in rural and hard-to-reach communities to enhance COVID-19 vaccination program in Ghana. Again, since the acceptability of the COVID-19 is low among these communities, people must have maintain related practices such as the social distancing and wearing of nose masks [23].

In this study, we observed that receiving recent or previous vaccine such as HBV vaccine, having good attitude towards COVID-19 vaccine and having good perception about the COVID-19 vaccine were independent predictors of higher chances of COVID-19 vaccine acceptability. These findings are in line with studies of Wang et al. [21], and Schmid et al. [24], who reported history of Influenza vaccination is a significant predictor of vaccine acceptability, and individual's attitudes and perceptions toward vaccines. This implies previous reviews and experiences are associated with individuals trust in health authorities and concurrently with infectious disease vaccinations. Individuals tend to rely on credible information and guidance from past and present to make a firm decision. Interestingly, in our study, some main reasons for unwillingness to accept vaccination were vaccine safety and its seroprotection. Therefore, a transparent, robust, and strategic immunization process can improve public confidence in the COVID-19 vaccine and future vaccines.

Moreover, we found low knowledge level about the COVID-19 vaccine among more than half of the study participants. This is similar to studies of Dereje et al. [25], and Islam et al. [26], who also found low knowledge level among Ethiopians and Bangladeshis respectively. However, our findings are lower compared to study by Al-Qerem et al. [27] who found more than two-thirds of Jordanians having adequate knowledge about COVID-19 vaccine. The lower knowledge level observed in our study maybe due to our study that centered on people living in hard-to-reach communities in Ghana. These communities consist of illiterates, with lower access to vital educational facilities and technology. This finding calls for the need to reach such communities with educational campaigns and awareness of the important of vaccination program.

Furthermore, in consistence with our findings of poor attitudes and perceptions towards COVID-19 vaccine is the study by Dereje et al. [25], and Mesesle et al. [28], who also found similar low attitude and perceptions towards COVID-19 vaccine. Again, the poor attitudes towards COVID-19 vaccine and bad perception about COVID-19 vaccine maybe attributed to low educational level among our study participants with majority of participant having no or basic educations. Mahmood et al. have recommended further awareness campaigns and knowledge of safe interventions to combat the spread of disease [29]. Educational interventions hammering on the role, safety and usefulness of the vaccines should be strategically implemented to greatly improve vaccine acceptability, attitude and perception in the Ghanaian population.

This study was limited by the fact that it was conducted during the time of vaccination roll out which some people may have heard or had some education about vaccination. Again, the incorporation of media awareness has impact on the dynamics of COVID-19vaccine acceptability and the observed results may vary in subsequent studies. Our study findings however highlight the fact that, acceptability, knowledge and perception about COVID-19 vaccine is still low among residents in rural communities in Ghana.

## Conclusion

More than half of residents in Ghanaian rural communities have inadequate knowledge, poor attitudes and bad perception about COVID-19 vaccine. The acceptability of COVID-19 vaccine is generally low among rural residents in Ashanti, Northern and Western North regions of Ghana. Residents living in hard-to-reach communities must be educated about the benefits of COVID-19 vaccine to achieve effective vaccination program. Previous vaccination experience has a role in the acceptability of COVID-19 vaccine and therefore national vaccination programs like the Hepatitis B program should be leveraged to promote COVID-19 vaccine acceptability.

## Data Availability

All data generated or analyzed during this study are included in this article and raw data can be requested from corresponding author.

## Abbreviations

CHRPE: Committee on human research, publication and ethics
COVID-19: Coronavirus infectious disease 2019
GPS: Ghana postal addressing system
HBV: Hepatitis B virus
SARS-CoV-2: Severe acute respiratory syndrome coronavirus-2
SMS: School of medical sciences
SPSS: Statistical package for social science
WHO: World Health Organization
